# Cellulose Acetate Microparticles Synthesized from *Agave sisalana* Perrine for Controlled Release of Simvastatin

**DOI:** 10.3390/polym16131898

**Published:** 2024-07-02

**Authors:** Larissa Pereira Alves, Kevin da Silva Oliveira, Ana Cláudia Gonçalves dos Santos, Demis Ferreira de Melo, Lívia Maria Coelho de Carvalho Moreira, João Augusto Oshiro Junior, Dayanne Tomaz Casimiro da Silva, Airlla Laana de Medeiros Cavalcanti, Bolívar Ponciano Goulart de Lima Damasceno

**Affiliations:** 1 Graduate Program of Pharmaceutical Sciences, Paraíba State University, Campina Grande 58429-600, PB, Brazil; larissaapereira@hotmail.com (L.P.A.); oliveira.kevinf@gmail.com (K.d.S.O.); demiscz@gmail.com (D.F.d.M.); carvalholivia312@gmail.com (L.M.C.d.C.M.); joaooshiro@yahoo.com.br (J.A.O.J.); dayannecasimiro@hotmail.com (D.T.C.d.S.); bolivarpgld@servidor.uepb.edu.br (B.P.G.d.L.D.); 2 Laboratory of Development and Characterization of Pharmaceutical Products, Department of Pharmacy, Paraíba State University, Campina Grande 58429-600, PB, Brazil; anaclaudia204@gmail.com

**Keywords:** microencapsulation, cellulose, controlled release, new drug release systems, bioavailability

## Abstract

Simvastatin (SIM) is widely prescribed to treat hyperlipidemia, despite its limitations, such as a short half-life and low oral bioavailability. To overcome these drawbacks, the development of a controlled-release formulation is desirable. This study aims to develop a microparticulate system based on cellulose acetate (ACT) obtained from *Agave sisalana* Perrine to promote a controlled SIM release. SIM-loaded microparticles (SMP) were prepared using the solvent emulsification-evaporation method. Several parameters were evaluated, including particle size, surface charge, morphology, encapsulation efficiency, thermochemical characteristics, crystallinity, and in vitro release profile. ACT exhibited favorable flow properties after acetylation, with a degree of substitution values superior to 2.5, as confirmed by both the chemical route and H-NMR, indicating the formation of cellulose triacetate. The obtained SMP were spherical with an average size ranging from 1842 to 1857 nm, a zeta potential of −4.45 mV, and a high SIM incorporation efficiency (98%). Thermal and XRD analyses revealed that SIM was homogeneously dispersed into the polymeric matrix in its amorphous state. In vitro studies using dialysis bags revealed that the controlled SIM release from microparticles was higher under simulated intestinal conditions and followed the Higuchi kinetic model. Our results suggest that ACT-based microparticles are a promising system for SIM delivery, which can improve its bioavailability, and result in better patient compliance.

## 1. Introduction

Statins are among the most widely used lipid-lowering agents due to their potential to reduce morbidity and mortality in cardiovascular diseases. Over the past decade, some researchers have demonstrated the anti-inflammatory, antioxidant, and neuroprotective properties of statins, which can be explored for potential benefits in central nervous system disorders, degenerative diseases, and various types of cancers [1,2].

Simvastatin (SIM) holds considerable importance for the pharmaceutical industry, boasting annual sales surpassing USD 3 billion, representing about 30% of the global sales volume of hypercholesterolemia medications [3,4]. This drug is a potent inhibitor of 3-hydroxy-3-methyl-glutaryl-coenzyme A (HMG-CoA) reductase, an enzyme responsible for converting HMG-CoA into mevalonate, a key precursor in cholesterol synthesis. According to the biopharmaceutical classification system, SIM is classified as a class II drug. In addition, it exhibits a short half-life (~2 h) and low oral bioavailability (<5%) due to its insufficient in vivo solubility and extensive first-pass metabolism [5,6].

In this context, several chemical or physical strategies can be employed to overcome these limitations, including the reduction in the size of the active pharmaceutical ingredient (API), prodrug development, co-crystal formation, salt formation, and the SIM encapsulation within microstructured systems. Among these approaches, the microstructured delivery systems stand out. These systems are formed by particles with sizes ranging from 1 to 1000 µm which can have the ability to regulate and extend the active pharmaceutical ingredient release. This last property is especially important once it can reduce the medication administration frequency contributing to better patient compliance [7,8].

The use of appropriate components to form the drug polymeric-based delivery system is the essential key to developing a stable system. Consequently, biodegradable polymers are the most commonly used materials due to their crucial characteristics, including low toxicity, good stability, biodegradability, high water permeability, elevated glass transition temperature, and compatibility with diverse pharmaceutical active ingredients [9,10].

Cellulose acetate (ACT) is one of the main organic esters derived from cellulose. This polymer has commercial importance based on its widespread use in the production of films, fibers, and plastic goods. Moreover, other characteristics can be highlighted such as its non-toxicity, cost-effectiveness, good processing performance, great biodegradability, and biocompatibility. Therefore, studies on the biotechnological production of polymeric materials from alternative raw material sources have gained increasing importance, as this technology can be less harmful to the environment and contribute to sustainable development [11,12,13].

Based on this fact, a way to optimize the use of *Agave sisalana* Perrine leaves could involve extracting the cellulose contained within them and subsequently synthesizing cellulose acetate polymer. This process would add value to agricultural waste by transforming it into consumer goods through the incorporation of this polymer in modified release systems, facilitating a connection between small rural producers and the pharmaceutical industry, leading to the economic development of the region and overall sustainability.

Therefore, the aim of this study was to develop a stable microparticle system formed by the cellulose acetate polymer synthesized from the cellulose presented in *Agave sisalana* Perrine and the stabilizing agent polyvinyl alcohol (PVA) for the purpose of SIM incorporation and the improvement of its characteristics. This work also included the characterization of the physicochemical properties and the investigation of the SIM release profile through in vitro analyses.

## 2. Materials and Methods

### 2.1. Materials

Cellulose acetate was synthesized from cellulose extracted from the *Agave sisalana* Perrine plant. The following chemicals were used in the study: acetone, acetic acid, sulfuric acid, nitric acid, ethyl alcohol, acetic anhydride, potassium biphthalate, dichloromethane, and potassium sodium hydroxide, all of which were supplied by Neon^®^, São Paulo, Brazil. Acetonitrile was obtained from Êxodo Cientifica^®^, Sumaré, SP, Brazil. PVA (MW: 13,000–28,000 kDa), hydrochloric acid, sodium chlorite, and phenolphthalein were supplied by Sigma-Aldrich, Milan, Italy. SIM was provided by Fragon, Guarulhos, SP, Brazil. Dialysis bags with a molecular weight cutoff of 12,000 Da were purchased from Sigma-Aldrich, Saint Louis, MO, USA. All the other chemicals were used as received without further processing.

### 2.2. Vegetable Raw Material

Samples of *Agave sisalana* Perrine were collected at the experimental farm of the Brazilian Agricultural Research Corporation (EMBRAPA) in Monteiro, PB, Brazil, at a latitude of 7°52′40.50′′ and a longitude of 37°07′34.91′′. The plant’s leaves were properly cleaned with running water, cut into sections, and submitted to dehydration in an air circulation oven maintained at a controlled temperature of 60 °C for 12 days, until a constant weight was achieved. Subsequently, the material was crushed in a knife mill equipped with four fixed and four movable blades, coupled to a 20 mm diameter sieve.

### 2.3. Cellulose Extraction Process I: Elimination of Soluble Compounds

The methodology used for the purification of crude *Agave sisalana* was the modified ethanol/nitric acid method, previously described by Rodrigues-Filho and colleagues (2007) [14], which is based on the oxidation of lignin by nitric acid. Briefly, 40 g of *Agave sisalana* were subjected to reflux with three successive portions of a 20% *v/v* mixture of nitric acid and ethanol. At each hour, the reaction mixture was changed, and the material was washed with distilled water. After 3 h of reflux, the mixture was filtered and washed repeatedly with distilled water until the washing solution became colorless. Following that, the material was immersed in sodium hydroxide solution (NaOH, 1 mol/L) for 24 h. After this period, the mixture was washed and neutralized using a 10% acetic acid solution. The *Agave sisalana* fibers were subsequently dried in an oven at 105 °C for 3 h and then the dried material was subjected to another batch of processing in a knife mill.

### 2.4. Cellulose Extraction Process II: Obtention of Holocellulose

Holocellulose is the product resulting from lignin extraction composed of cellulose and hemicelluloses. The process of lignin removal utilizes sodium chlorite as the main reagent and relies on the reaction between lignin and ClO_2_/ClO^−^, which are formed after the redox reactions of ClO_2_ in an acid medium. In brief, to obtain holocellulose, 5.0 g of the crude extract of *Agave sisalana* Perrine was weighed, and 100 mL of distilled water was added. The mixture was heated in a water bath at 75 °C, followed by the addition of 0.5 mL of acetic acid and 0.75 g of sodium chloride. This procedure was repeated twice, with the addition of 0.5 mL of acetic acid and 0.75 g of sodium chlorite each hour. After three hours of reaction, the mixture was cooled to 10 °C, filtered, and washed with distilled water at 5 °C until the fibrous residue became whitish. Finally, the fibrous residue was dried at 105 °C for 3–6 h and stored in a sealed desiccator until further use [15,16].

### 2.5. Extraction of Cellulose

For this process, 10.0 g of holocellulose was transferred to a porcelain evaporating dish, and 100 mL of 24% KOH solution (*w:v*) was added to it. The mixture was stirred mechanically for 15 h at room temperature and then filtered through a glass crucible with a pre-weighed porous plate. The resulting solid residue was washed with two portions of 1% acetic acid and distilled water until the retentate was neutral followed by a final washing with ethanol. The obtained cellulose was dried at room temperature on glass plates [15].

### 2.6. Production Cellulose Acetate

In order to obtain the cellulose acetate, 1.0 g of *Agave sisalana* cellulose was mixed with 25 mL of glacial acetic acid under stirring. This mixture was stirred at 500 rpm for 30 min at room temperature (25 °C). Afterward, a solution containing 0.08 mL of concentrated sulfuric acid (H_2_SO_4_) and 9.0 mL of glacial acetic acid was added and stirred for an additional 25 min at room temperature. Following this step, the mixture was filtered and then 32 mL of acetic anhydride was added to the filtrate. The combined solution was stirred with a glass rod and returned to the original flask with the material. It underwent further stirring at 500 rpm for an additional 30 min before being allowed to settle. After 14 h, distilled water was gradually added to the reaction medium until no more precipitate formed. Finally, the material was dried in an oven for 90 min at 105 °C [17,18].

### 2.7. Determination of the Yield of the Extraction Process

The cellulose acetate yield was calculated using the method described by Santos and colleagues (2016) [19], which involves weighing both the initial material (crushed and screened *Agave sisalana* Perrine) and the final product in an analytical balance, corresponding to the purified cellulose acetate. The below Equation (1) was applied to determine the cellulose acetate yield:

(1) $$Y\left(\%\right)=\frac{Wfinal}{Winicial}\times 100$$

where: Y(%) = yield in percentage; Wfinal = final weight of the obtained polymer; Winitial = initial weight of crushed and screened *Agave sisalana.*

### 2.8. Determination of the Degree of Substitution of Cellulose Acetate

#### 2.8.1. Chemical Route

The degree of substitution (DS) was obtained through a saponification reaction. Briefly, 0.1 g of cellulose acetate was added to 5.0 mL of sodium hydroxide (0.25 mol/L) and 5.0 mL of ethanol. This mixture was allowed to rest for 24 h. After this period, 10 mL of hydrochloric acid (0.25 mol/L) was added to the mixture, and it was left to stand for an additional 30 min. The solution was then titrated using sodium hydroxide, which was standardized with potassium phthalate, with phenolphthalein as the indicator. This procedure was conducted in triplicate.

Determination of the DS by the chemical route is based on the percentage quantification (% m/m) of the acetyl groups (AG) that were replaced by hydroxyl groups in the cellulosic chain. In simpler terms, the DS (Equation (2)) is calculated based on the AG value obtained using Equation (3).

(2) $$DS=\frac{162\times GA}{43\times 100-42\times GA}\times 100$$

where AG are calculated using Equation (3):

(3) $$AG=\frac{\left[Vb\times µb-Va\times µa\right]43\times 100}{mac}$$

where Vb represents the total volume of sodium hydroxide used during the procedure, including both the amount added and the amount consumed in the titration, Va corresponds to the volume of hydrochloric acid added, µa and µb denote the molarities of the hydrochloric acid and sodium hydroxide solutions, respectively, and mac represents the mass of sodium acetate used.

#### 2.8.2. 1H-NMR Spectrometry

The 1H-NMR spectra were obtained from cellulose acetate solutions derived from the *Agave sisalana* extraction in deuterated chloroform in the proportion of 20 mg·g^−1^ (polymer/solvent). Spectra were recorded at 50 °C on a Varian Unity 500 NMR spectrometer using a 5 mm probe and a frequency of 500 MHz. Standard acquisition parameters included a 45° RF pulse, an interpulse repetition time of 8 s, and 32,000 points covering a spectral width of 7200 Hz. The DS value determined by the H-NMR method was calculated from the ratio of the area corresponding to the resonance of the hydrogens presented in the glycosidic rings (Agly) and the area under the hydrogens of the methyl group (ACH_3_) according to Equation (4):

(4) $$DS=\frac{7{ACH}_{3}}{3Agly}$$

### 2.9. Microparticles Preparation

SIM-loaded microparticles (SMP) were prepared using the solvent emulsification-evaporation method. SIM was dissolved in a solution consisting of 0.10 g of cellulose acetate within 15 mL of dichloromethane (DCM) to produce particles with a concentration of 10:1 m/m (acetate:SIM). The organic phase solution (acetate/SIM/DCM) was prepared and it was subsequently added drop by drop into 100 mL of an aqueous solution containing 1.5% polyvinyl alcohol (PVA) under vigorous stirring (5000 rpm). DCM was removed by evaporation through a bench spray dryer, model MSD 0.5 (LABMAQ^®^, Ribeirão Preto, SP, Brazil).

### 2.10. Physicochemical Characterization

#### 2.10.1. Particle Size, Polydispersity Index, Zeta Potential and Encapsulation Efficiency

In order to obtain the average particle size, polydispersity index (PDI), and zeta potential, photon correlation spectroscopy and electrophoretic light scattering were employed through a Zetasizer (Brookhaven’s ZetaPals instrument, New York, NY, USA). Prior to analysis, the samples were appropriately diluted with deionized water in a 1:1 ratio at a constant temperature of 25 °C.

The SIM concentration incorporated into SMP was determined by UV-Vis spectrophotometric analysis (Shimadzu^®^ UV-1900, Japan). In brief, approximately 2.0 g of SMP were dissolved in 0.5% sodium lauryl sulfate (SLS) to obtain solutions at 1 mg/mL concentration. These solutions were centrifuged (Hettich^®^ Centrifuge—MIKRO 220 R, Germany) at 11,000 rpm for 30 min to determine the amount of free drug. Total SIM concentration was extracted from the SMP by complete microparticle dissolution in SLS after 48 h of agitation.

The drug concentration was quantified by measuring the absorbance of SIM at 238 nm using ultraviolet-visible spectrophotometry (UV-Vis) (Shimadzu, UV-1900, Japan). Various parameters, such as selectivity, linearity, precision, accuracy, detection limit (LD), the limit of quantification (LQ), and robustness were determined according to the well-established guidelines of the Agência Nacional de Vigilância Sanitária (ANVISA) (Supplementary Materials). The calibration curve was constructed according to the SIM concentration range of 3 to 30 μg/mL and the encapsulation efficiency (EE%) of SMP was calculated using Equation (5):

(5) $$EE\%=\frac{Amount\;of\;encapsulated\;drug}{Total\;amount\;of\;drug}\times 100$$

(6) $$Drug\;Loading\;efficiency(\%)=\frac{Microparticle\;drug\;concentrarion}{Total\;mass\;of\;the\;formulation}\times 100$$

#### 2.10.2. Scanning Electron Microscopy (SEM)

Microparticle surface and morphology were assessed using a Zeiss^®^ scanning electron microscope (model LEO 1430). Samples were distributed on metal specimen holders (stubs) with double-sided carbon adhesive tape. They were then sputter-coated with gold for 3 min using a vacuum coating device (Emitech k550x, Ashford, England, UK). Subsequently, they were directly analyzed at a 15 kV accelerating voltage.

#### 2.10.3. Thermal Analysis

Thermogravimetry (TG) and derivative thermogravimetry (DTG) curves of the isolated components, their binary mixtures, and the microparticles were obtained with a simultaneous thermobalance module TG/DTA, model Q600 (TA Instruments, New Castle, DE, USA), using alumina crucibles with about 4 ± 0.1 mg under a nitrogen atmosphere at a flow rate of 50 mL min^−1^, a temperature range from 25 to 900 °C, and a heating rate of 10 °C min^−1^. The equipment was calibrated with calcium oxalate monohydrate.

The DSC curves were obtained on a TA Instruments calorimeter, model DSC Q20 (TA Instruments, New Castle, DE, USA), using hermetically sealed aluminum crucibles with about 2 ± 0.05 mg of samples. The rising temperature experiments were conducted under a nitrogen atmosphere at a flow rate of 50 mL min^−1^, with a temperature range from 25 to 300 °C, and a heating rate of 10 °C min^−1^. Indium (melting point 156.6 °C) was used as the standard for equipment calibration. Data were analyzed using the software TA Instruments Universal Analysis 2000, 4.5A.

#### 2.10.4. X-ray Diffraction Analysis (XRD)

X-ray diffraction patterns of SMP and their individual solid components were analyzed using a powder X-ray diffractometer (XRD-6000 Shimadzu^®^, Kyoto, Japan) operating on 40 kV and a current of 40 mA, employing a θ/2θ geometry with scanning from 10° to 80° (0.02° s^−1^) and Cu (Kα1) radiation. The data were plotted by means of the software OriginPro version 8.5.

#### 2.10.5. Fourier Transform Infrared Spectroscopy (FT-IR)

FT-IR analysis was performed using a Spectrum TM 400 FT-IR/FT-NIR spectrometer (Perkin Elmer^®^, Boston, MA, USA) with a resolution of 4 cm^−1^, scanning speed of 0.2 cm^−1^ ranging from 4000 to 650 cm^−1^.

#### 2.10.6. In Vitro SMP Release

The in vitro SIM release was evaluated using the dialysis membrane (DM) method, employing simulated gastric (pH 1.2) and intestinal fluid (pH 6.8) as the release medium. A formulation amount equivalent to 5 mg of SIM was placed on a dialysis membrane with a molecular weight cutoff of 12,000 Da (Sigma-Aldrich^®^). This membrane was immersed in 100 mL of the release medium maintained at 37 ± 0.5 °C with constant stirring at 100 rpm. Samples were collected at predefined time intervals of 15 min, 30 min, 45 min, 1 h, 2 h, 4 h, 6 h, 8 h, 12 h, 18 h, 24 h, 48 h, and 72 h. The medium was replaced with an equal volume of fresh medium each time to ensure the sink conditions. The collected samples were analyzed to determine the released SIM concentration using a UV-Vis spectrophotometer at 238 nm (Shimadzu^®^ UV-1900, Japan).

#### 2.10.7. Statistical Analysis

All results were expressed as the mean ± standard deviation (SD) from three independent repetitions. The significance of differences between groups was determined using one-way analysis of variance (ANOVA) followed by Tukey’s multiple comparison tests, performed using OriginPro 8.5 2019 (Northampton, MA, USA), with a significance level set at *p* < 0.05.

## 3. Results

### 3.1. Extraction of Cellulose and Cellulose Acetate from Agave sisalana Perrine

**Figure 1:**
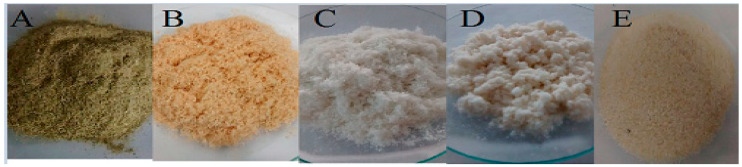
Technological processing of the plant drug to cellulose acetate. Photographs: (**A**) vegetable drug, (**B**) plant drug free of extractives (purified), (**C**) holocellulose, (**D**) cellulose, and (**E**) cellulose acetate.

The yield of the cellulose extraction process from the plant material was 19.0432 ± 1.3475%. The cellulose acetylation reaction aimed to produce ACT yielded 83.0631 ± 3.9%. All the stages of the purification and bleaching processes are represented in Figure 1. Steps A to B show the purification of crude *Agave sisalana* by the modified ethanol/nitric acid method. The B to C phase characterizes the delignification process for obtaining holocellulose. Steps C to D exhibit the holocellulose components’ separation to obtain isolated cellulose, while the D to E phase represents the cellulose acetylation process.

### 3.2. Degree of Substitution of Cellulose Acetate

#### 3.2.1. Chemical Route

Cellulose acetate obtained from *Agave sisalana* cellulose exhibited a DS value of 2.72 ± 0.27 and an AG percentage of 42.26% ± 2.54, which corresponds to a characteristic value for this biopolymer.

#### 3.2.2. H-NMR

**Figure 2:**
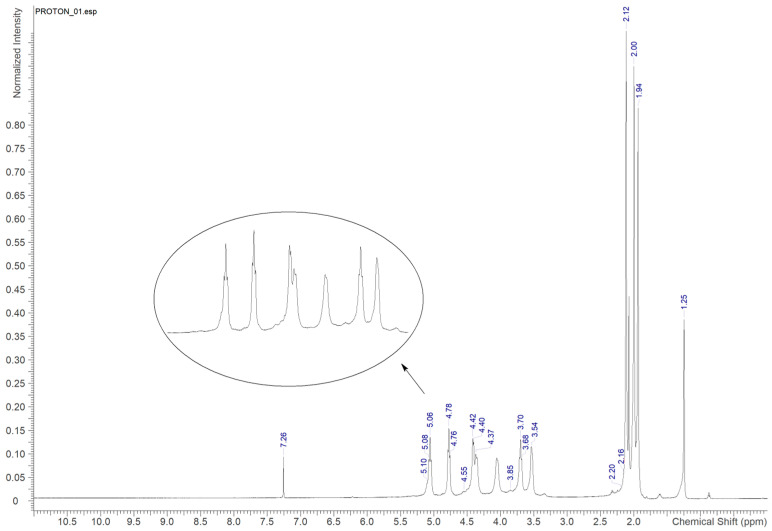
1H-NMR spectra of cellulose acetate.

H-NMR spectrum for cellulose acetate is illustrated in Figure 2. A singlet signal was observed at 7.26 ppm corresponding to the solvent peak (CHCl_3_). The structural analysis presented two clusters of hydrogen signals: the resonance signals of the glucose ring (3.53–5.10 ppm), showing seven distinctly resolved proton resonances attributable to the 2,3,6-triacetylated AGUs, and the resonance corresponding to methyl protons of the acetate group (1.23–2.19 ppm). The DS was calculated from these chemical shifts using the proton NMR method, resulting in a value of 3.49.

### 3.3. Characterizations of Microparticles

#### 3.3.1. Morphological Analysis

**Figure 3:**
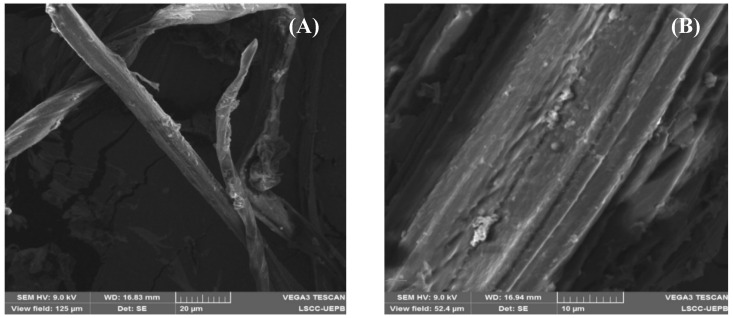
Photomicrographs of cellulose obtained by SEM. (**A**) 200× magnification. (**B**) 500× magnification.

**Figure 4:**
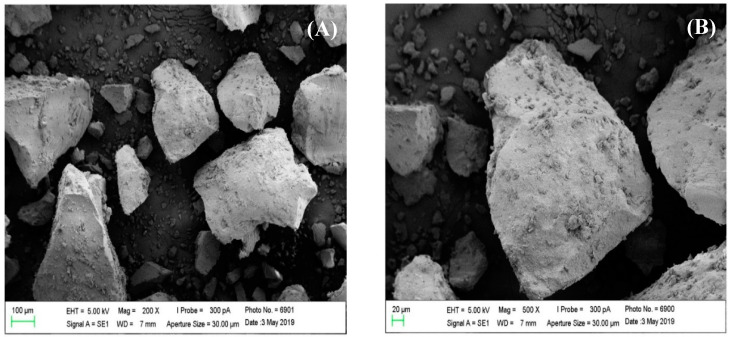
Photomicrographs of cellulose acetate obtained by SEM. (**A**) 200× magnification. (**B**) 500× magnification.

**Figure 5:**
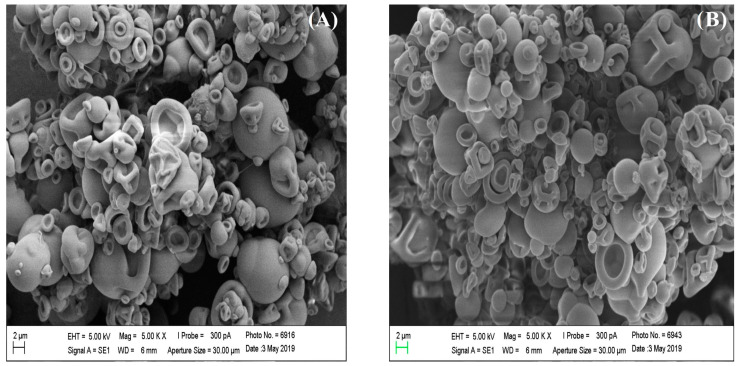
Photomicrographs of microparticles obtained by SEM. (**A**) Microparticle without simvastatin—5000× magnification. (**B**) SIM-loaded microparticles—5000× magnification.

The shape and surface morphology of the cellulose and ACT obtained from *Agave sisalana* were accessed by SEM. Visual representations of cellulose and ACT can be observed in Figure 3 and Figure 4, respectively. The SEM images of cellulose and ATC exhibited distinct characteristics. Cellulose presented a network-like structure with a fibrous surface, typical of lignocellulosic materials. Following the acetylation process, the fibrous characteristic was lost, and ACT showed a structurally smooth appearance with irregular shapes and dimensions, coupled with a slight roughness on its surface. SEM analyses of the BMP and SMP were also performed, and the resulting images are visualized in Figure 5. Both types of microparticles showed an evidently spherical shape, smooth surface with minimal roughness, and a diverse range of sizes. Additionally, the particles presented an aggregation tendency and the presence of deformities in certain microparticles, further contributing to the overall complexity of the samples.

#### 3.3.2. Particle Size, Polydispersity Index, Zeta Potential and Encapsulation Efficiency

The physicochemical properties of the blank (BMP) and optimized SMP are shown in Table 1. The particle sizes ranged from 1842 to 1857 nm for BMP and SMP, respectively. PDI results were similar between the microparticles, with values in the range of 0.2. Microparticles containing SIM presented a zeta potential of −4.45 ± 1.12 mV, whereas for the BMP, the value was −3.79 ± 0.26 mV. Regarding encapsulation efficiency, the optimized SMP exhibited a SIM incorporation rate of 98.92 ± 2.6%, suggesting the successful inclusion of the drug into the system. The Drug Loading value (DL), which represents the amount of drug incorporated into the nanoparticles, was calculated as 6.2% by weight, indicating efficient encapsulation of the drug in the delivery system.

#### 3.3.3. Thermal Analysis

**Figure 6:**
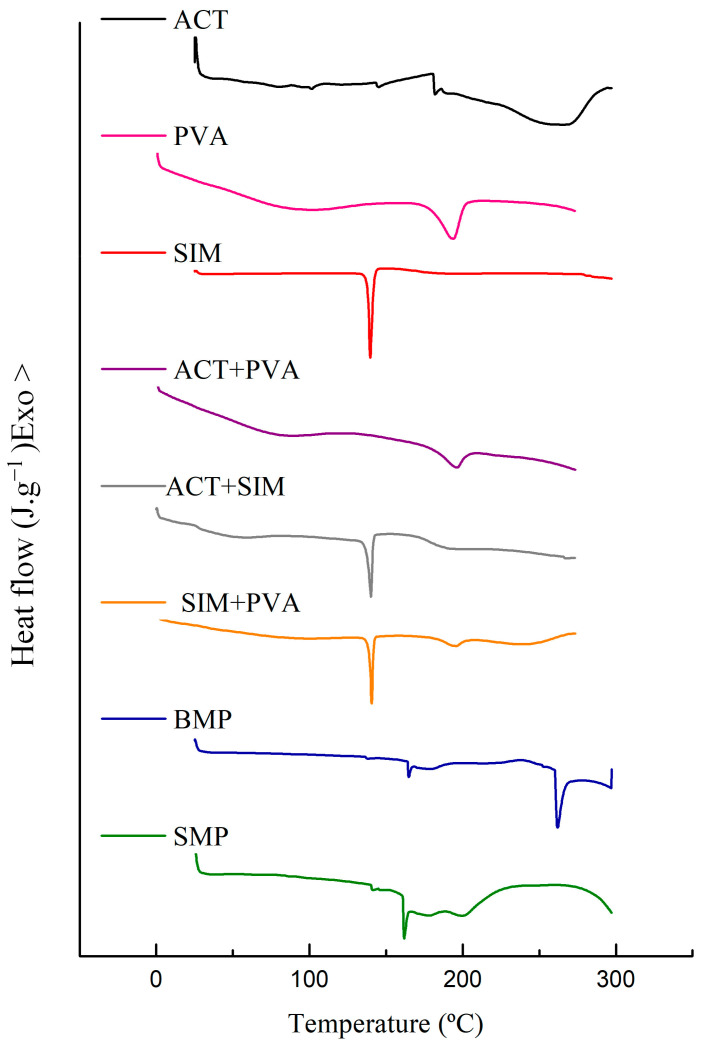
DSC curves of the cellulose acetate (ACT), polyvinyl alcohol (PVA), simvastatin (SIM), the binary mixtures [cellulose acetate+polyvinyl alcohol (ACT+PVA), cellulose acetate+simvastatin (ACT+SIM), and simvastatin+polyvinyl alcohol (SIM+PVA)] and microparticles without the drug (BMP) and with simvastatin (SMP).

**Figure 7:**
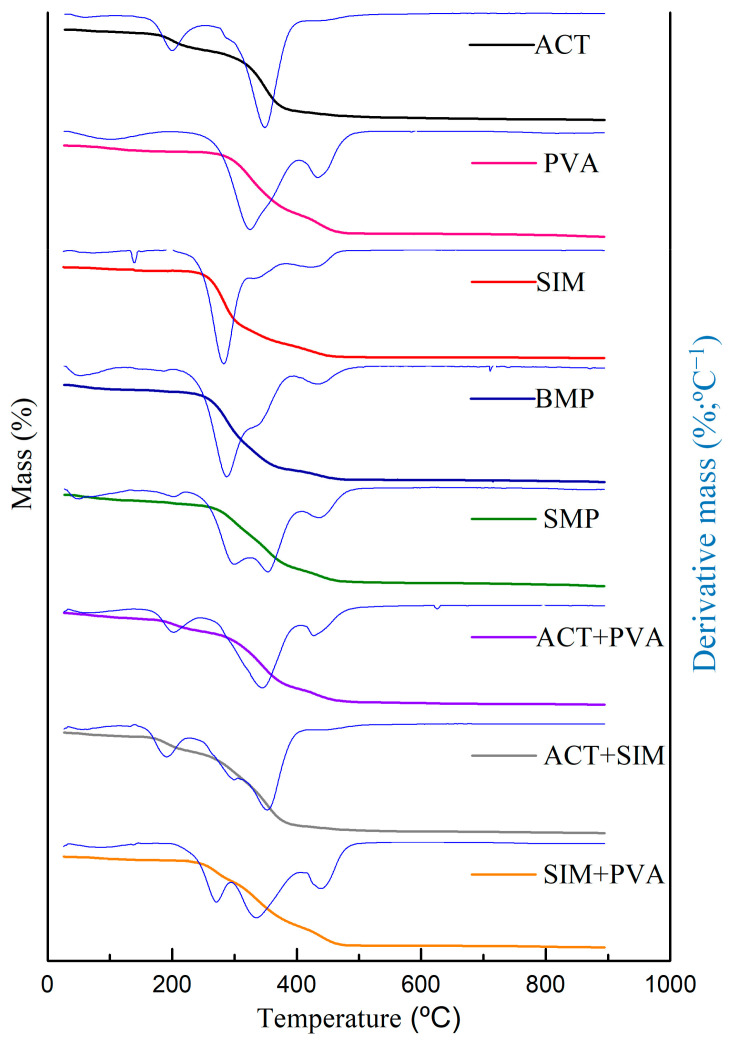
Simultaneous TGA/DTG thermograms of cellulose acetate (ACT), polyvinyl alcohol (PVA), simvastatin (SIM), the binary mixtures [cellulose acetate+polyvinyl alcohol (ACT+PVA), cellulose acetate+simvastatin (ACT+SIM), and simvastatin+polyvinyl alcohol (SIM+PVA)] and microparticles without the drug (BMP) and with simvastatin (SMP).

The DSC and TG/DTG curves of ACT, PVA, SIM, binary mixtures, and microparticles obtained can be observed in Figure 6 and Figure 7, respectively. Both additional TGA data for excipients and binary mixtures and DSC heating curves and temperature data can be found in Supplementary Material.

The DSC curve for ACT shows a first endothermic peak at 144.18 °C (ΔH = −0.2529 J g^−1^) associated with the desorption of water molecules bound to the structure of the cellulosic derivative, which was confirmed by the slight mass loss of 4.33% verified in the range of 31–214 °C in the TG/DTG curves. The glass transition temperature of the polymer can be observed at 181.82 °C. The noted exothermic peak at 186 °C (ΔH: +0.3890 J g^−1^) can be associated with the polymer crystallization process. The substantial endothermic event occurred between 220 and 300 °C (ΔH: −25.18 J g^−1^), with a peak at 268.82 °C, which is indicative of the gradual degradation process of the polymer, corroborated by the most significant mass loss (Δ_m%_ = 72.18%) in the TG/DTG curve, which occurred between 214 and 402 °C. The last step in the ACT TG curve, whose temperature was between 402 and 605 °C, indicated a 22.48% mass loss.

Analyzing the DSC data for the PVA, two endothermic events were identified. The first peak detected at 98.53 °C (ΔH = −41.30 J g^−1^) characterizes the polymer dehydration, which is reiterated with the first event presented in the TG/DTG curve which occurred between 39 and 224 °C, resulting in a mass loss of 6.82%. The second endothermic peak at 194.16 °C (ΔH = −40.08 J g^−1^), can be attributed to the melting point of semicrystalline PVA. Two more distinct stages of degradation occurred in the PVA thermogravimetric curve. The step between 224 and 397 °C led to a mass loss of 70.8%. Finally, the third stage, whose temperature was between 397 and 560 °C, indicated a 22.35% mass loss.

The DSC curve of pure SIM exhibited a single prominent endothermic event at 139.62 °C (ΔH = −49.81 J g^−1^) suggestive of its crystalline drug melting point. Subsequently, after the melting phase, the SIM degradation process began, confirmed by a substantial mass loss (69.89%) within the temperature range of 198–330 °C, as depicted in the TG curve. Additional thermogravimetric events were apparent, with the first one occurring at 330–382 °C and the second at 382–483 °C, resulting in mass losses of 14.15% and 12.25%, respectively.

In the case of the ACT+PVA mixture, two distinct endothermic events were observed, highlighting the one occurring at 78.05 °C (ΔH = −9.90 J g^−1^) attributed to the dehydration of the polymers. This moisture loss is supported by the TG event presented at the temperature range of 31–155 °C, leading to a mass loss of 6.40%. Interestingly, the melting temperature of the PVA component remained relatively constant at 179.76 °C (ΔH = −11.36 J g^−1^). Additionally, two significant TG events were detected. The most substantial mass loss (Δ_m%_ = 60.15%) occurred between 245 and 408 °C, indicating the degradation of both ACT and PVA. The final step, observed in the range of 408–570 °C, is associated with the main degradation event of PVA and resulted in a 16.45% mass loss.

The DSC curves of the binary mixtures ACT+SIM and PVA+SIM presented characteristic endothermic peaks of the SIM melting. These peaks were observed at 137.15 °C (ΔH = −22.64 J g^−1^) and 139.09 °C (H = −17.47 J g^−1^), respectively, indicating the preservation of the drug’s crystalline state. In the TG curves for the ACT+SIM mixture, the thermal decomposition process was characterized by five distinct stages. The principal mass losses occurred during the second (138–228 °C), third (228–309 °C), and fourth (309–427 °C) steps, leading to 14.49%, 23.27%, and 47.68% of mass losses, respectively. In the case of the PVA+SIM mixture, the most important decomposition event started at the temperature range of 181–296 °C, resulting in a mass loss of 37.8%, resembling the SIM behavior.

Regarding the DSC analysis of SMP, no distinct peak corresponding to the melting of SIM was observed in the curve. This lack of a phase transition suggests that the loaded SIM existed in an amorphous state. The TG decomposition profiles of BMP and SMP showed quantitative differences. The principal thermal event for BMP occurred at the temperature range of 205–327 °C, resulting in a mass loss of 56.00%. In contrast, for SMP, the onset of the main degradation event shifted to a higher temperature range between 224 and 326 °C, accompanied by a lower mass loss (Δ_m%_ = 37.15%).

#### 3.3.4. Fourier Transform Infrared Spectroscopy

**Figure 8:**
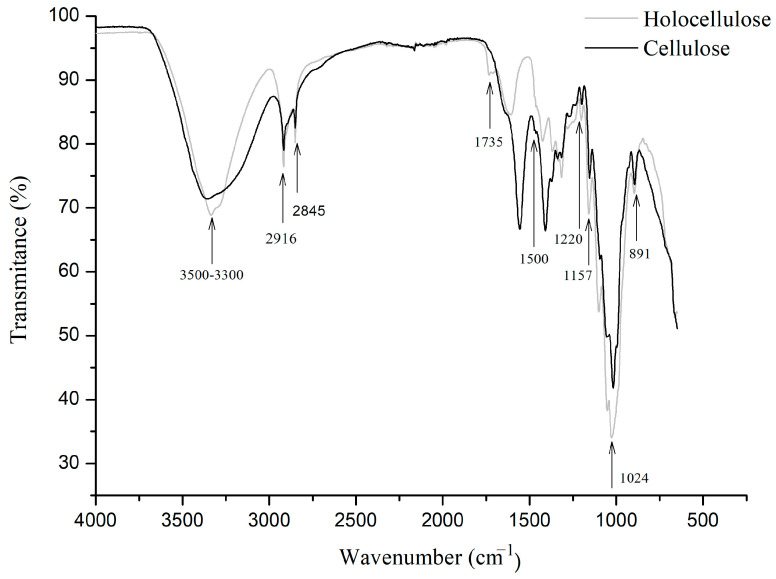
FTIR spectrum for holocellulose and cellulose.

Through the main infrared spectral differences, it is possible to identify changes in the cellulosic fiber and assign the characteristic absorbance bands of crystalline cellulose I and II. Figure 8 presents the results of FTIR spectra on the characteristic structure of holocellulose and cellulose. As bands at 3500–3300 cm^−1^ correspond to hydroxyl (O-H) stretching vibration, bands of medium intensity are C-H stretching characteristics of the sp3 carbon observed at 2916 cm^−1^. At 1735 cm^−1^, it corresponds to the acetyl and uronic ester group or the ester bond of the carboxylic group of hemicellulose. However, it is possible to announce that after the alkaline treatment, the disappearance of this band in the cellulose spectrum was obtained, confirming the successful removal of hemicellulose after the chemical treatment. At 1500 cm^−1^, they are attributed to the C=C vibration of aromatic lignins, while at 1220 cm^−1^ they are attributed to the C=O stretching of lignins. At 1024 cm^−1^, they are attributed to C-O vibrations of hemicelluloses and 893 cm^−1^ for the C-O-C elongation of the B(1-4) glycoside bond typical of cellulose, and are attributed to amorphous cellulose. Compared to holocellulose, the intensity of the cellulose spectrum and the intensity of absorption are high, which suggests increased amounts of cellulose [1].

Nelson and collaborators [2] proposed that the ratio of intensities in the FTIR study can be analyzed by the crystallinity index. Consequently, the crystallinity study focuses on determining the spectral ratios 1420/893 and 1375/2902 cm^−1^ which can be applied to both types of cellulose (I and II) or to a mixture of both components. In the case of samples predominantly composed of crystalline cellulose II, the ratio 1420/893 cm^−1^ (lateral order index, LOI) increases with a decreasing degree of crystallinity. Likewise, the 1375/2900 cm^−1^ (total crystalline index, TCI) is proportional to the degree of crystallinity of the cellulose samples. At 1375 cm^−1^, it is attributed to a C-H curvature mode, whereas the band at 2900 cm^−1^ is attributed to C-H elongation. Therefore, the ratio of holocellulose and cellulose intensities is shown in Table 2.

**Figure 9:**
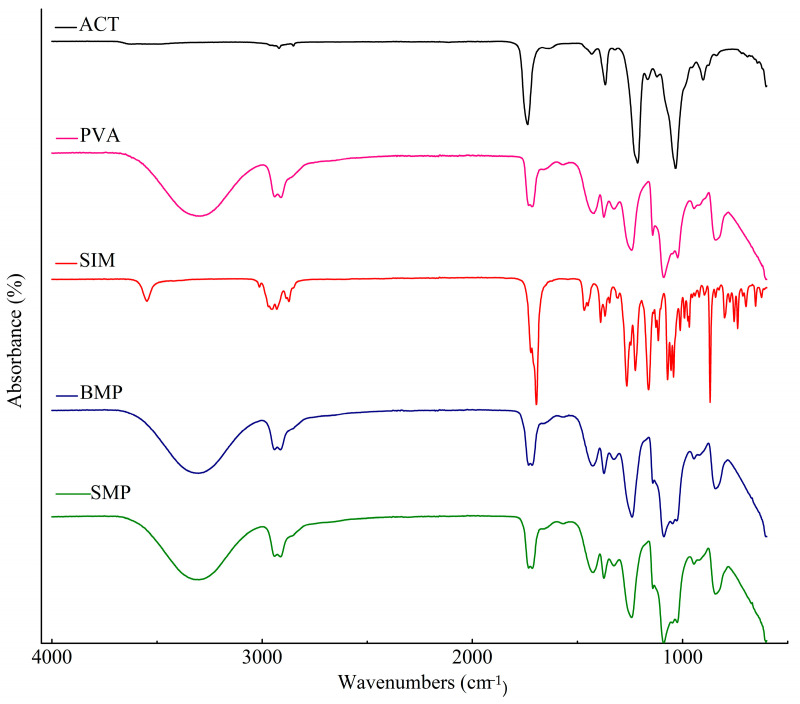
FTIR spectrum for cellulose acetate (ACT), polyvinyl alcohol (PVA), simvastatin (SIM), microparticle without the drug (BMP), and microparticle with simvastatin (SMP).

The absorption spectra in the infrared region of ACT, PVA, SIM, and microparticles are shown in Figure 9. Moreover, Table 2 presents the assignments of the main bands regarding the absorption spectra.

ACT exhibited low-intensity bands between 3700 and 3200 cm^−1^, attributed to the stretching of residual hydroxyl groups. An intense band at 1745 cm^−1^ corresponds to the stretching of the C=O bond from the ester group present in the acetyl group structure. The rise of the band at 1368 cm^−1^ corresponds to C-H bond vibrations. Additionally, an intense band at 1035 cm^−1^ is attributed to C-O bond vibration, which plays a crucial role in the bonding between cellulose monomers. The last band is at 1033 cm^−1^, associated with pyranose ring bond vibrations (C-O-C) and the acetyl group.

For PVA, a typical absorbance intensity at 3305 cm^−1^ was attributed to the OH group. An acute band at 2945 cm^−1^ denotes the CH stretching of alkyl groups, while the bands at 1424 and 1373 cm^−1^ indicate CH vibrations. The carboxylic (C=O) stretching can be observed at 1722 cm^−1^. Additionally, bands at 1095 and 838 cm^−1^ correspond to C-O stretching from alcohol groups.

The SIM FTIR spectrum showed a specific band at 3548 cm^−1^ as a result of the OH stretching vibrations of alcohol. At 3008 cm^−1^ there is a CH olefinic stretch, and at 2937 and 2875 cm^−1^, we can observe an asymmetric and symmetric stretching CH vibration of methyl and methylene groups. Bands at 1700 and 1694 cm^−1^ were associated with the stretch of the lactone C=O and ester C=O, respectively. Furthermore, the bands at 1468 and 1392 cm^−1^ correspond to methyl and methylene C-H bending vibrations, while at 1273 cm^−1^ there is a lactone and ester C-O-C bending vibrations. At 1073 cm^−1^, there is the secondary alcohol C-O stretching, and finally, at 870 cm^−1^, a C-H vibration of the trisubstituted olefin (Table 3). The infrared spectra of BMP and SMP exhibited a broad similarity, indicating no significant differences and a substantial overlap of the curves of its constituents. In the SMP curve, the characteristic drug absorption bands were absent, suggesting the SIM encapsulation within the microparticles.

#### 3.3.5. X-ray Diffraction

**Figure 10:**
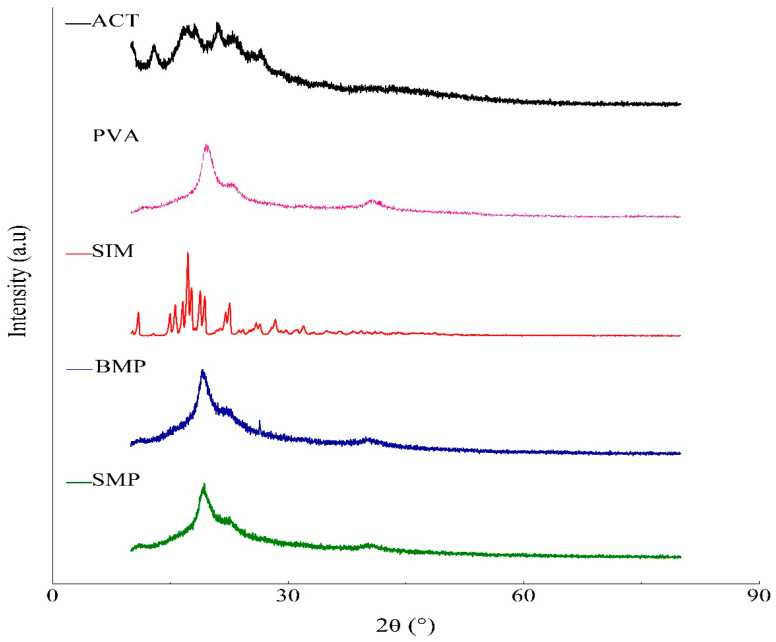
XRD pattern for cellulose acetate (ACT), polyvinyl alcohol (PVA), simvastatin (SIM), microparticle without the drug (BMP), and microparticle with simvastatin (SMP).

The crystallinity of ACT, PVA, SIM, and SMP was evaluated using XRD, the most suitable technique for detecting this characteristic (Figure 10). The ACT diffractogram exhibited a broad halo at 2θ = 20°, characteristic of amorphous regions. Reflections near 2θ = 10° and 12.76° were also noted and indicated a shift in peak position, demonstrating the cellulose acetylation. For PVA polymer, wide semi-crystalline reflections were observed, predominantly at 2θ = 19.5°, 22.7°, and 40.7°. The SIM X-ray diffractogram showed multiple diffraction peaks at 2θ = 10.9°, 13°, 14.92°, 15.58°, 16.54°, 17.32°, 17.7°, 18.88°, and 19.36°, characteristic of the crystalline nature of the drug. Dominant reflections (doublet) are also evident at approximately 17.5°, corresponding to the polymorphic form I. Conversely, in the SMP X-ray diffractogram, the SIM characteristic acute peaks disappeared, suggesting the dispersion of the drug in the matrix system. Additionally, a higher intensity reflection appeared at 2θ = 19.34°, which is associated with the organization of polymer chains.

#### 3.3.6. In Vitro SMP Release

**Figure 11:**
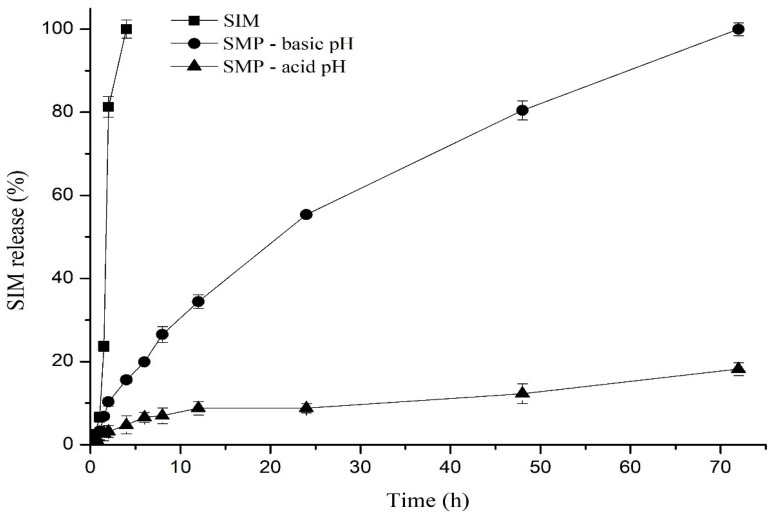
In vitro release profiles of SIM from SMP in simulated gastric (pH 1.2) and intestinal fluids (pH 6.8) over a 72 h period at 37 °C. The data are expressed as mean ± SD (n = 3).

The in vitro release profile of SIM over a period of 72 h is represented in Figure 11. In contrast to free SIM, which exhibited a sudden release pattern characterized by continuous and increasing release, reaching its maximum release percentage within the initial period, about 2 h, even before being exposed to the simulated intestinal pH. In the first 5 h of the experiment, the release of SIM from the microparticles was a controlled process, extending throughout the entire 72 h period without an explosion effect. Variations in the receiving medium influenced the release of SIM from microparticles. For example, in the first two hours, approximately 10.28% and 3.21% of SIM were released into the basic and acidic medium, respectively.

The selection of the most suitable mathematical kinetic model to describe the SIM release from the microparticles was determined through a linear regression analysis of the obtained data. Table 4 presents the mathematical models applied to the SIM release profile. Comparisons of determination coefficients (R^2^) revealed that the SIM release profile from the microparticles followed Higuchi’s kinetic model. Furthermore, the periodic SLS changes effectively prevented the accumulation of SIM in the dissolution medium, ensuring that it did not exceed 10% of its saturation level. The release profile maintained a steady-state flow rate of 468 µg/√h, and it took just 1.71 h to achieve a constant release rate.

## 4. Discussion

The increasing demand for pharmacological therapies that enhance drug efficacy, improve its pharmacokinetic standards, and present greater accessibility, coupled with the discourse to explore sustainable alternatives in the pharmaceutical industry, drove our research group to develop a microparticulate system based on ACT polymer derived from *Agave sisalana* for use as a controlled release system for SIM. The encapsulation of this drug within microparticles can lead to better therapeutic performance by facilitating controlled release and, thus, prolonging its effect in the body. This fact becomes particularly crucial in the context of long-term treatments, in which stable plasma drug levels are essential to achieve satisfactory clinical results.

The average yield of the cellulose extraction from *Agave sisalana* was successfully estimated in this work. According to previous studies, various methodologies were employed for polymer extraction to determine cellulose content. Nevertheless, this approach yielded values that were close to the estimated cellulose content percentages for agricultural residues, which typically range from 20 to 60% [3,4].

The cellulose acetylation reaction resulted in a significant synthesis of cellulose acetate with a yield higher than the results presented in the study of Amaral and colleagues (2019) [5]. These authors achieved a 76% yield using the same methodology of synthesis to obtain cellulose from the babassu coconut endocarp. Our results were also superior to those shown by Goswami and colleagues (2019) [6] who attained a yield of 74% *w/w* using a different type of catalyst in the reaction to obtain cellulose acetate from *Alpinia nigra*, an herb belonging to the ginger family.

Following the obtainment of cellulose acetate, we determined its DS, an important parameter defined as the average number of hydroxyl groups present in a cellulose anhydroglucose unit that has been esterified with acetyl groups. Each anhydroglucose unit contains three free hydroxyl groups attached to carbon 2, 3, and 6; therefore, material with different DS can be obtained. The DS value can range from zero, as seen in cellulose, to three, as in cellulose triacetate, thereby exerting an influence on the solubility, crystallinity, and biodegradability of the polymer according to the value [7,8].

Our DS results are in accordance with the previous studies of Brites and colleagues (2020) [9] showing that a DS value exceeding 2.5 is indicative of cellulose triacetate. We obtained similar DS values between the chemical route and the H-NMR analysis, and both were superior to 2.5, suggesting a probable production of ACT. In addition, elevated DS values are achieved when high lignin solubilization occurs in the extraction process. The lignin must be reduced to a minimum amount once this compound competes with cellulose for the acetylation reactants, and consequently a high content of lignin can potentially decrease the acetylation capacity of cellulose [10,11]. Therefore, the DS values obtained in this study also confirm the effective lignin solubilization and removal during the extraction procedure.

In sequence, we decided to investigate the morphology and surface characteristics of cellulose, ACT, BMP, and SMP through SEM analyses. As expected for a lignocellulosic material, the cellulose image exhibited a network-like and fibrous pattern, a characteristic previously mentioned in studies about these materials [12]. This intrinsic aspect of cellulose was substantially affected in the ACT images, leading to notable changes in the surface morphology, shape, and dimensions. The morphological alteration can be attributed to the acetylation process, once the hydroxyl groups of glucose units were substituted with acetyl groups. Prior to acetylation, these hydroxyl groups participated in inter- and intramolecular hydrogen bonding, playing a crucial role in the formation of the intricate network structure of cellulose [5,6].

The morphological analysis of the microparticles, both with or without SIM, revealed important characteristics, such as the spheric format, a range of sizes, deformities, and agglomeration. The spherical configuration of the formulations facilitates the powder flow, and the various sizes of smaller particles enable them to fit within the interstices of larger ones, thus promoting an efficient particle arrangement [13]. This size variation could occur due to the active electrostatic forces acting on the microparticles during the spray-drying process, as described by Li and colleagues (2010) [14]. Most of these microparticles exhibited aggregation, compromising the material flow promoted by the spherical shape. This phenomenon can also be attributed to the spray-dryer drying process or a specific step during the emulsification method, similar to other studies with polymeric systems using alginate, gum Arabic, and maltodextrin [15,16,17]. Some particles also exhibited more wrinkled surfaces, which can be related to a rapid drying process. According to Ye and colleagues (2019) [18], when the drying process is fast, the droplets undergo accelerated heat and mass transfers, potentially leading to deformities in the microparticles’ surfaces. This same structural phenomenon can also be observed in the microparticulate systems developed by Guerreiro and colleagues [19].

The physicochemical properties of the microparticles are detailed in Table 1. Despite the broad range of sizes observed in the microscopic analysis, the evaluations of size and PDI indicated that the microparticles exhibited a narrow size distribution, describing the uniformity of the system and suggesting that a majority of the particles share similar dimensions. These data reinforce the efficiency of the employed method to obtain homogeneous particle sizes, as evidenced by the PDI values obtained below 0.3. Such values are generally considered indicative of a monodispersed distribution and uniform diameters of particulate systems [20]. Zeta potential is an important indicator of the surface potential that determines the repulsion magnitude in the electrical double layer, which influences the physical stability of various colloidal systems. BMP and SMP exhibited values of zeta potential close to each other. These lower modular values can indicate that the polymeric layers presented in the microparticles shifted the shear plane to a greater distance from the particle surface, resulting in low zeta potentials [21], which can also contribute to the aggregation phenomenon mentioned before. SMP exhibited a notable drug EE%. Our value was superior to those obtained by other studies utilizing SIM encapsulated in microparticles formed by different polymers, such as chitosan and polylactic-co-glycolic acid [22,23], as well as studies employing similar experimental methodologies for microparticle production [19,24,25]. Therefore, this substantial EE% value represents a crucial parameter to be considered since it can potentially lead to reductions in net weight or the final required dose volume.

Considering the characterization data obtained, we performed DSC and TG/DTG analyses to assess the compatibility and thermal stability of the individual substances, their binary mixtures, and the resulting microparticles. In compatibility studies, thermoanalytical techniques allow for the rapid preselection of more stable pharmaceutical formulations through the evaluation of potential interactions that may exist in their binary mixtures [26,27,28].

The DSC curve for ACT shows an initial endothermic peak associated with the outflow of water molecules bound to the structure of the cellulosic derivative [29], a phenomenon consistent with observations in the thermogravimetric curve that attributed its first slight mass loss to the evaporation of the volatile compounds and the release of water bound to the hydrophilic (OH) groups within the ACT chains, consequently leading to ACT deacetylation [30,31,32,33,34]. The glass transition temperature of the polymer occurred within the same temperature range reported in the other studies [35]. The ACT exothermic peak was associated with the polymer crystallization process [35]. The most prominent endothermic event in the DSC curve is also related to the most significant mass loss in the TG result, revealing the polymer degradation process through the pyrolytic decomposition of the polymeric ACT chain. This process involves the cleavage of glycosidic bonds, as well as the decomposition of lignin and hemicellulose chains [6,36]. The final TG event corresponds to the carbonization process, resulting in the complete degradation and decomposition of the polymer.

The thermal analysis of PVA revealed two distinct endothermic events in the DSC curve. The first event is associated with the evaporation of the physically absorbed water molecules, a phenomenon also observed in a previous study [37]. This event is further confirmed by the initial degradation step in the TG curve. The second endothermic peak can be attributed to the melting point of semicrystalline PVA. Upon completing the TG analyses, three mass loss events were observed, similar to those obtained by previous studies [38,39]. The second mass loss event resulted from the elimination of acetic acid to form the polyene, and the final mass loss event is attributed to the main ruptures of the PVA chain, as previously reported by [40,41].

The sharp peak identified at the DSC curve of SIM is characteristic of the melting point of the crystalline drug. According to The Merck Index (2013) [42], SIM crystals should exhibit a melting point within the range of 135–138 °C. Nevertheless, other studies employing DSC with the same heating rate (10 °C/min) have suggested a slightly broader range between 136 and 140 °C for the SIM melting point. These variations in temperature measurements can be attributed to differences in heating rates and the specific equipment employed during the analysis, as highlighted in studies by [43,44]. No crystalline transition event was observed, and the initial absence of melting point duplication excluded the presence of polymorphs in SIM [43,44]. Therefore, based on the obtained data, the purity level of SIM was estimated at approximately 99.45%, suggesting a high degree of purity. Following the melting phase, the thermal decomposition of SIM occurred, as depicted in the TG curve. The most substantial mass loss was attributed to the drug degradation.

The DSC curves of the binary mixtures PVA+SIM and ACT+SIM were performed to evaluate the potential interactions between the drug and the polymers. In both curves, we can observe the endothermic peaks characteristic of the SIM melting point, which remained well-defined and in the same temperature range. These data indicate the preservation of the crystalline state of the drug after mixing with the polymers. Therefore, we can suggest that no physical interaction occurred between the drug and excipients during the microparticle formation process. Regarding the ΔH (J g^−1^) of these mixtures, there was a reduction of approximately 50% in its value compared to pure SIM, once these are physical mixtures in a 1:1 ratio.

No characteristic melting peak of SIM was found in the DSC curve obtained from the SMP, closely resembling the thermal behavior of the polymers. The absence of the phase transition suggests that the encapsulated SIM is in its amorphous state, indicating that the drug is homogeneously dispersed into the polymeric matrix at the system molecular level [30,45]. These results are in agreement with those found by Webber and colleagues (2018) [31], who suggested that either the encapsulation process or the smaller proportion of drug to polymers used in microparticle production may have facilitated the amorphization of the drug. The conversion of SIM from a crystalline to an amorphous state is desirable since it contributes significantly to the drug dissolution rate and bioavailability [31].

Both the ACT produced from *Agave sisalana*-extracted cellulose and PVA exhibited infrared spectrum profiles that corresponded to the expected characteristics for these polymers [10,46,47,48,49,50]. For ACT, the low-intensity bands, attributed to the stretching of the remaining hydroxyl groups in the polymer chemical structure, result from the substitution of free OH groups by the acetate groups. These data serve as confirmation of the polymer DS when compared to the intensity of the carbonyl group absorption band [26,51,52,53]. All the characteristic absorption peaks of SIM were presented in the infrared spectrum and are in accordance with the literature [44,54,55].

The infrared spectrum characteristic bands of crystalline cellulose I (1430, 1162, and 1111 cm^−1^) were not found for the cellulose obtained, which indicates the predominance of crystalline cellulose II. Spectra in the range of 2800–3600 cm^−1^ are observed, and absorption bands at 3490 and 3448 cm^−1^ are typical of the intramolecular hydrogen bonds of –OH elongation present in crystalline cellulose II [56]. Furthermore, the most significant and most defined absorption band, at 893 cm^−1^, corroborates the absence of crystalline cellulose I. In this sense, we can state that the cellulose obtained is mainly composed of cellulose II. The type of cellulose obtained makes this allomorph the most thermodynamically stable form, due to an additional hydrogen bond per glucose residue [57]. The results obtained indicate that cellulose presents greater crystallinity compared to holocellulose. This fact is expected, since the chemical process modifies the morphological structure of the fibers, leading to the partial rupture of some crystalline regions for later reorganization into a different, but more stable polymorphic structure.

The profiles of BMP and SMP were very similar and there was a significant overlap in their characteristic peaks. In the case of microparticles containing SIM, the characteristic drug absorption bands either disappeared, shifted, or were replaced by polymer-related bands. This event suggests the entrapment of SIM within the polymeric matrix, with less exposure on the surface of microparticles. These alterations can be partially attributed to the low SIM content and the higher quantity of polymers in the system. Similar findings have been reported by other authors who incorporated the same drugs or other bioactive substances into their systems [45,58,59,60].

The ACT diffractogram analyses revealed a broad halo known as a van der Waals halo, which is characteristic of amorphous regions commonly found in organic polymers [30,61,62,63]. The reflections related to the semi-crystalline region of the ACT exhibit this reduced crystallinity due to the nearly complete disruption of intermolecular and intramolecular hydrogen bonds within cellulose during the substitution of hydroxyl groups by acetyl groups [64]. The presence of semi-crystalline reflections in the PVA X-ray diffractogram is a result of the strong intermolecular and intramolecular hydrogen bonds between PVA molecular chains [51,65,66].

The XRD pattern of pure SIM showed multiple intense and sharp peaks, confirming the crystalline nature of the drug. The occurrence of doublets in the SIM diffractogram corroborates the literature findings, which establish that polymorphic form I is the most stable solid form among the four identified to date [52,53]. Notably, all these SIM characteristic crystalline peaks are absent in the SMP diffractogram, indicating that the drug is molecularly distributed in the matrix system. This observation further confirms that SIM did not crystallize within the matrix [52]. Consequently, the combined results of DSC and XRD strongly suggest that SIM is homogeneously dispersed within the polymeric matrix in an amorphous state.

The in vitro drug release assesses the ability of the pharmaceutical system to release the drug, the amount of drug released over time, and the speed at which this phenomenon occurs [67]. Dialysis methods for determining the drug release profile have been widely employed in polymeric systems to guide formulation development, quality control, and establishing the in vitro-in vivo correlation of the formulation.

The release profile of the SIM incorporated into the microparticles was evaluated in both acidic (pH 1.2) and basic (pH 7.2) conditions at body temperature (37 °C), to simulate the gastric and intestinal pH, respectively, over a period of 72 h. The pure SIM was also used to obtain a release profile and to evaluate the free drug behavior under the same conditions. Due to the low aqueous solubility of SIM, SLS (0.5% *w/v*) was used as the receiver medium to dissolve the released SIM and maintain the sink conditions in the system. The reduced release of SIM from the microparticles in an acidified medium may be attributed to the ACT characteristics, once it contains carboxyl groups that remain protonated at acidic pH and, therefore, insoluble in stomach pH (1 to 3). Conversely, at small intestine pH (5.5 to 7), these groups ionize and become soluble. Consequently, drug release in the gastric region is limited, while a higher proportion of the drug might be completely released and absorbed in the intestine [68,69,70].

The selection of the best-fitting mathematical kinetic model to elucidate the mechanism underlying SIM release from the microparticles was based on the highest value of the R^2^ among the considered models, including zero-order, first-order, Higuchi, and Korsmeyer-Peppas. Consequently, due to its robust linearity, the Higuchi kinetic model best described the SIM release from the microparticles. This model establishes a linear correlation between the square root of time and the cumulative drug release, indicating that the drug release process is controlled by a diffusion mechanism in which the rate of drug transfer is directly proportional to the concentration gradient [71].

According to this model, the SIM release from microparticles involved the penetration of the release medium into the pores within the polymeric matrix, drug dissolution, subsequent drug diffusion through the pores, and finally the drug transfer to the release medium due to the initial difference in SIM concentration between the dialysis bag and the medium, which was the step that drove the drug passage to this solution [72,73].

The biodegradable nature of the polymers employed in drug-loaded microparticles, alongside the geometry and structure of the pore network, plays a pivotal role in the drug release process. This is due to their contribution to the diffusion of the drug on the surface of the pores, which form during the microencapsulation process or through the degradation of the polymer. In addition, the drug diffuses within the polymeric matrix itself, since the amount of drug solubilized in the polymer matrix and not covalently linked to it is related to the total amount of drug released [72,73].

The ACT microparticles utilized to encapsulate the SIM drug exhibited an encapsulation efficiency (EE%) of 98.92 ± 2.60%. Other polymers have been employed to facilitate the development of microparticles for SIM encapsulation, yet they have demonstrated a lower encapsulation efficiency. In a previous study by Zhang and colleagues, non-spherical polylactic-co-glycolic acid (PLGA) microparticles were developed for SIM encapsulation. This yielded particles with a lower encapsulation efficiency (EE%) of 48.8 ± 2.8% compared to spherical microparticles [74]. This was because non-spherical particles have a higher EE% than spherical structures due to their irregular shape. In the work carried out by Gentile et al. [75], PLGA microparticles were developed to encapsulate SIM, with an EE% of 92%. Masaeli et al. [76] obtained an EE% of 85%.

In addition, natural polymers offer economic advantages in the development of microspheres. An extensive market research report by BCC, entitled *“Microspheres:”*, is available. The report, *“Technologies and Global Markets”,* examined both the production and market perspectives on microspheres and found that the global market for these particles reached USD 3.8 billion in 2021. The report estimates that the market will reach a value of USD 5.2 billion by 2027 [77].

Consequently, natural polymers, such as cellulose derivatives, represent a cost-effective raw material with an average unit cost of EUR 790 per kilogram. These natural polymers are particularly appealing in particle synthesis due to their relatively low cost compared to synthetic polymers. Additionally, they are renewable, biocompatible, and biodegradable, exhibit excellent mechanical performance, and possess tunable surface chemistry [78]. In practice, the JNC Corporation markets spherical cellulose beads designated “CellufineTM”. These beads, which are purported to be effective for the purification of proteins, enzymes, and other biomolecules, represent an example of cellulose microparticles. The company Cytiva™ Technologies offers Cytopore™ macroporous microcarriers, which are cross-linked cotton cellulose particles with a diameter of 200 to 800 µm [79]. These are designed for use in suspension culture systems for the growth of cells and the production of recombinant proteins for therapeutic use [80].

The in vitro release test assesses the ability of the system to release the encapsulated drug, offering an indication of the system’s behavior [67]. Although this kind of study is only a premise for understanding the SMP performance, it was considered an adequate method to highlight the importance of the microparticle components and their microstructure in the SIM release through artificial membranes. This finding underscores the potential of the developed formulation as a promising system for controlled SIM release, which can lead to an improvement in the bioavailability of the drug and patient adherence to treatment.

## 5. Conclusions

In the present study, the pharmaceutical excipient ACT was adequately obtained from *A. sisalana*, thoroughly characterized, and employed in the production of SMP using the solvent emulsification-evaporation method. SIM exhibited a high encapsulation efficiency and the microparticles showed desirable characteristics regarding the distribution of sizes and morphology. The thermal behavior and diffractogram profile from SPM demonstrated the homogeneous SIM dispersion within the polymeric matrix, highlighting the system’s potential as a promising vehicle for encapsulating hydrophobic compounds. SIM released from the microparticles followed a kinetic model controlled by a diffusion mechanism and was more pronounced in a slightly acidic medium, reflecting the optimal environment for SIM delivery. Therefore, SMP can be a promising system capable of extending the therapeutic effect of SIM through controlled release behavior, thereby improving patient compliance to the treatment. Furthermore, these findings serve as a premise for the potential incorporation of other drugs that are required to be released in a controlled manner into this innovative system, which contains a key polymer derived from an alternative and sustainable source abundant in Brazilian soils.

## Figures and Tables

**Table 1:** Physicochemical properties of blank microparticles and SIM-loaded microparticles.

Parameters	BMP	SMP
Particle size (nm)	1842.74 ± 4.1	1857.36 ± 1.30
PDI	0.229 ± 0.042	0.278 ± 0.008
Zeta potential (mV)	−3.79 ± 0.26	−4.45 ± 1.12
EE (%) DL (%)	-	98.92% ± 2.60 6.2%

**Table 2:** Laterally determined infrared crystallinity indices (LOI) and total crystallinity (TCI).

Substrate	LOI (1420/893 cm^−1^)	TCI (1375/2900 cm^−1^)
Holocellulose	1.11	0.93
Cellulose	0.90	0.98

**Table 3:** The main bands (cm^−1^) observed in the FTIR spectra of the samples.

Samples	O-H	C-H	C-O	C=O	C-O-C
ACT	3552	2917	1035	1745	1033
PVA	3305	2945	1095	1722	-
SIM	3548	3008	1043	1700	1273
BMP	3299	2945	1087	1715	-
SMP	3309	2943	1087	1715	-

**Table 4:** Determination coefficient (R^2^) values obtained from fitting the simvastatin release profile to various mathematical models. Qt—amount of drug released at time t; Q_0_—initial amount of drug in solution; Mt—amount of drug dissolved at time t; M∞—total amount of dissolved drug when the nanoparticle fully disintegrates; K, K_1_, KH—diffusion constants specific to each model; t—time.

Kinetic Model	Equation	Line Equation	Determination Coefficient (R^2^)
Zero-order	Q_0_ = Q_t_ + K_0_.t	y = 1.5957x + 8.4063	0.9784
First order	ln Qt = ln Q_0_ + K_1_.t	y = 0.0171x + 1.0522	0.6958
Higuchi	Qt = K_H_.t^1/2^	y = 14.66x − 12.807	0.9917
Korsmeyer-Peppas	Mt/M∞ = K.t^n^	y = 0.8194x + 1.436	0.9752

## Data Availability

Data are contained within the article and Supplementary Materials.

## Supplementary Materials

The following supporting information can be downloaded at: https://www.mdpi.com/article/10.3390/polym16131898/s1, Figure S1. Scanning spectrum from 190 to 400 nm, with three absorption peaks, with a wavelength of 238 nm corresponding to the maximum absorption of the drug; Figure S2. Scan spectrum of BMP and SMP showing the formation of the characteristic peaks of the drug. Figure S3. Analytical curve of simvastatin solutions in 0.5% sodium lauryl sulfate in concentrations of 3 to 30 µg/mL; Table S1. Analysis of variance in regression (ANOVA); Table S2. Angular coefficient significance test; Table S3. Analytical values of the precision of the validated spectrophotometric method (n = 6); Table S4. Analytical values of the accuracy of the validated spectrophotometric method (n = 3); Table S5. Analytical values of the robustness of the validated spectrophotometric method (n = 3); Table S6. Thermal decomposition results through thermogravimetry analysis. Cellulose acetate (ACT), polyvinyl alcohol (PVA), simvastatin (SIM), microparticle without drug (BMP), and microparticle with simvastatin (SMP); Table S7. Results of the calorimetric events of the samples. Cellulose acetate (ACT); polyvinyl alcohol (PVA), simvastatin (SIM), microparticle without drug (BMP) and microparticle with simvastatin (SMP). Figure S4. Holocellulose and cellulose DSC curve.
