# Supplementary material for: Cellulose Acetate Microparticles Synthesized from Agave sisalana Perrine for Controlled Release of Simvastatin
Source: Polymers (Basel). 2024 Jul 2;16(13):1898. doi: 10.3390/polym16131898 (PMC11243862; doi:10.3390/polym16131898)

Supplementary material

Figure S1. Scanning spectrum from 190 to 400nm, with three absorption peaks, with a wavelength of 238 nm corresponding to the maximum absorption of the drug

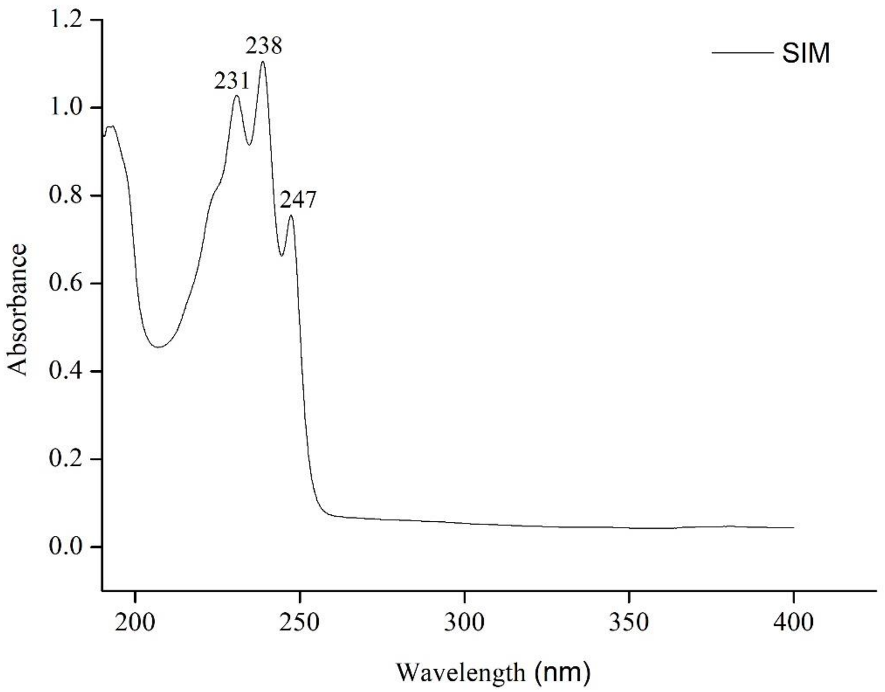

Figure S2. Scan spectrum of MPSF and MPCF showing the formation of the characteristic peaks of the drug.

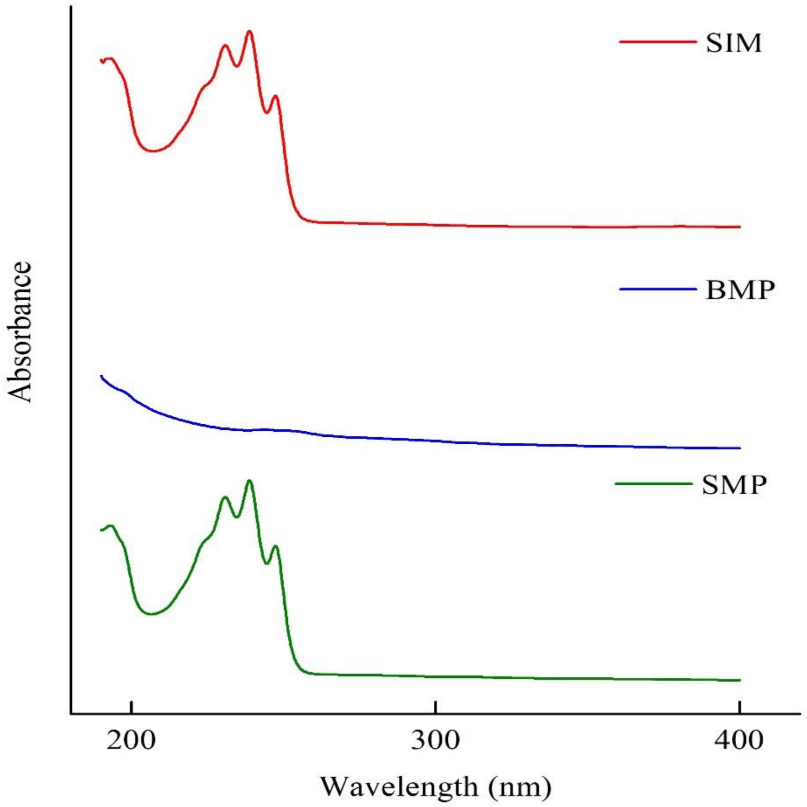

Figure S3. Analytical curve of simvastatin solutions in 0.5% sodium lauryl sulfate in concentrations of 3 to 30 µg / ml.

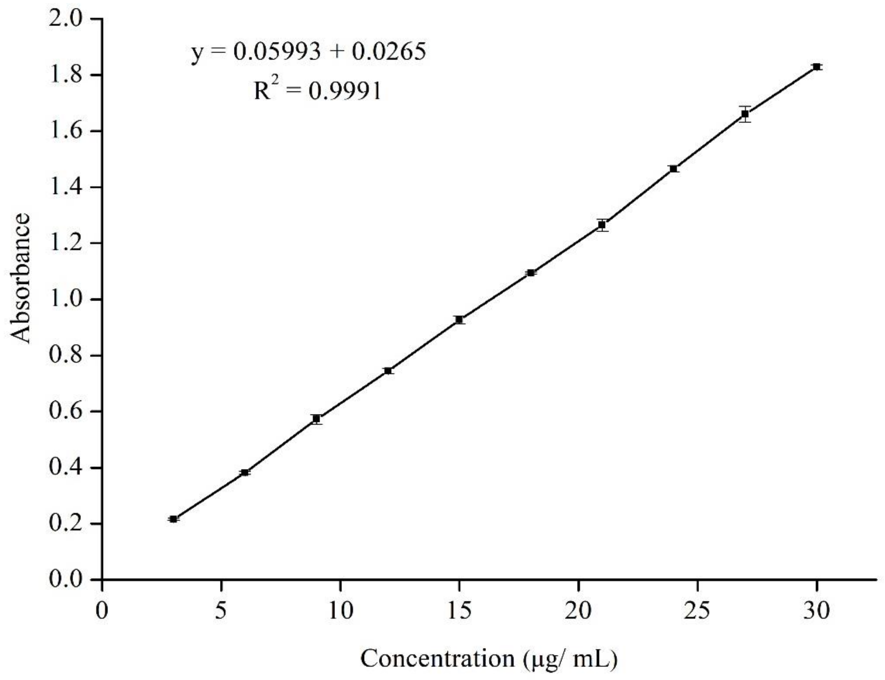

Table S1. Analysis of Variance in Regression (ANOVA).

	DF	QS	QA	F
Regression	1	8.0011899	8.0011899	$F_{1,28}$ 31284.90875
Residual	28	0.007161067	0.000255752	-
Lack of fit	8	0.003173733	0.000396717	$F_{8,20}$ 1.989884635
Pure error	20	0.003987333	0.000199367	-
Total	29	8.008350967	-	-

Legend: QS = Quadratic Sum. DF = Degrees of Freedom. QA = Quadratic mean.

Table S2. Angular coefficient significance test

	Coefficients	Pure error	Statistic t	Value-p	95% low	95% high
Intersection	0.026466	0.006307	4.196108	0.000248	0.013546	0.039387
Variable X	0.059933	0.338845	176.8754	3.12E-44	59.23924	60.62743

Table S3. Analytical values of the precision of the validated spectrophotometric method (n = 6).

Analysts	Day	Concentration	Mean absorbance	SD	CV (%)
Analyst 1	1	15 µg/mL	0.9277	0.0119	1.2785
	2	15 µg/mL	0.9480	0.0327	3.4518
Analyst 2	1	15 µg/mL	0.9568	0.0247	2.5833
	2	15 µg/mL	0.9760	0.0127	1.3057

SD = standard deviation; CV = coefficient of variation.

Table S4. Analytical values of the accuracy of the validated spectrophotometric method (n = 3).

Level	Mean absorbance	SD	CV (%)	Theoretical concentration	Real concentration	Accuracy (%)
Low	0.2093	0.0025	1.2022	3 µg/mL	3.04 µg/mL	101.55%
Medium	0.9426	0.0105	1.1143	15 µg/mL	15.28 µg/mL	101.91%
High	1.8637	0.0235	1.2611	30 µg/mL	30.66 mg/mL	102.21%

SD = standard deviation; CV = coefficient of variation.

Table S5. Analytical values of the robustness of the validated spectrophotometric method (n = 3)

Modification	Mean absorbance	Theoretical concentration (µg/mL)	Real concentration ± SD	Precision CV (%)	Accuracy (%)
pH 1.4	0.9270	15	0.0150 ± 0.024	2.6693	100.0742
pH 7.4	0.9363	15	0.0151 ± 0.009	0.0090	101.1073

SD = standard deviation; CV = coefficient of variation.

Table S6. Thermal decomposition results through thermogravimetry analysis. Cellulose acetate (ACT), polyvinyl alcohol (PVA), simvastatin (SIM), microparticle without drug (BMP) and microparticle with simvastatin (SMP).

Samples	Events	Temperature range (°C)	Mass (%)
ACT	1	31-214	4.335
	2	214-402	72.182
	3	402-605	22.485
PVA	1	39-224	6.82
	2	224-397	70.80
	3	397-560	22.35
SIM	1	38-145	3.61
	2	198-330	69.83
	3	330-382	14.15
	4	382-483	12.25
ACT + PVA	1	31-155	6.40
	2	155-245	12.31
	3	245-408	60.15
	4	408-570	16.45
SIM + ACT	1	40-138	4.04
	2	138-228	14.49
	3	228-309	23.27
	4	309-427	47.68
	5	427-578	8.00
SIM + PVA	1	39-144	4.30
	2	181-296	37.8
	3	296-408	37.0
	4	408-568	19.72
,BMP	1	31-121	5.00
	2	121-205	2.47
	3	205-318	56.00
	4	318-398	27.71
	5	398-515	8.76
SMP	1	32-134	7.82
	2	134-224	4.52
	3	224-326	37.15
	4	326-408	40.76
	5	408-510	8.75

Table S7. Results of the calorimetric events of the samples. Cellulose acetate (ACT); polyvinyl alcohol (PVA), simvastatin (SIM), microparticle without drug (BMP) and microparticle with simvastatin (SMP).

Samples	Events	T_{peak} (ºC)[a]	ΔH ($J.g^{-1}$)[b]
ACT	1	144,18	0.2529
	2	181,82	0.8845
	3	186,00	0.3890
	4	268,82	25.18
PVA	1	98,53	41.30
	2	194,16	40.08
SIM	1	139,62	49.81
ACT + PVA	1	78,50	9.903
	2	179,76	11.36
ACT + SIM	1	50,36	19.64
	2	137,15	22.64
	3	190,17	17.38
SIM + PVA	1	139,09	17.47
	2	182,74	11.91
	3	249,22	36.86
BMP	1	164,78	2.338
	2	261,88	25.37
SMP	1	140,92	0.5217
	2	161,76	3.591

a peak temperature
b heat enthalpy

Figure S4. Holocellulose and cellulose DSC curve.

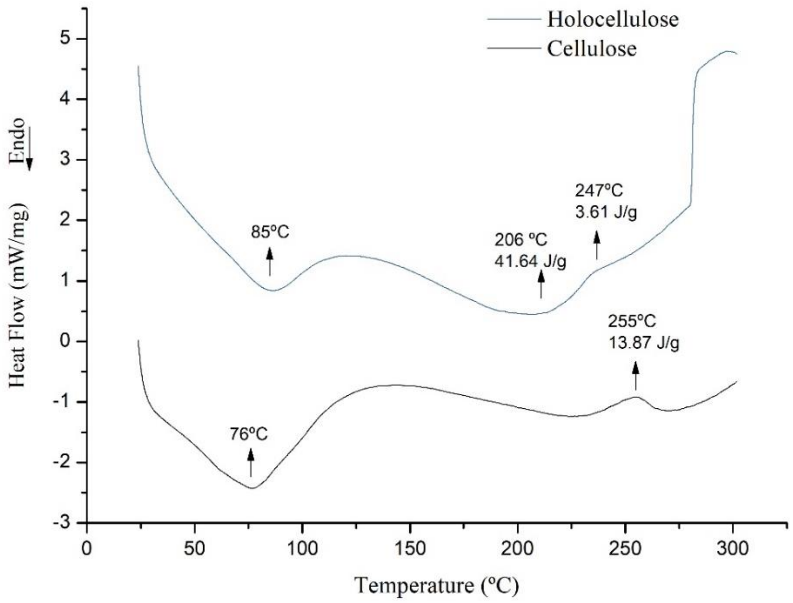

Supplement: Supplementary file 1 [file polymers-16-01898-s001.zip › polymers-3039749-supplementary.pdf]
